# Task‐Related Neurotransmitter Dynamics in the Visual Cortex: A Proof‐of‐Concept Time‐Resolved fMRS Study Using a Sustained Vigilance Paradigm

**DOI:** 10.1002/nbm.70389

**Published:** 2026-09-08

**Authors:** I‐Ting Lee, Shang‐Hua N. Lin, Rong‐Rung Kuo, Ching‐Po Lin, Li‐Hung Chang

**Affiliations:** ^1^ Institute of Neuroscience National Yang Ming Chiao Tung University Taipei Taiwan; ^2^ Center for Healthy Longevity and Aging Sciences National Yang Ming Chiao Tung University Taipei Taiwan; ^3^ Department of Education and Research Taipei City Hospital Taipei Taiwan; ^4^ Brain Research Center National Yang Ming Chiao Tung University Taipei Taiwan

**Keywords:** fMRS, Glx, neurochemistry, sustained attention, time‐resolved analysis, visual cortex

## Abstract

The neurochemical mechanisms underlying sustained visual processing, particularly in the early visual cortex, remain unclear. Earlier research has primarily focused on higher‐order frontal and parietal regions, yet emerging evidence suggests that the visual cortex may contribute to early‐stage sustained visual processing. Functional magnetic resonance spectroscopy (fMRS) provides a unique opportunity to detect task‐induced changes in neurometabolite concentrations, enabling dynamic assessment of neurometabolic processes. In this proof‐of‐concept study, five participants, all female, with a mean age of 26.5 ± 4.0 years, underwent three fMRS sessions on consecutive days using a sustained vigilance paradigm targeting the visual cortex. Time‐resolved analyses were applied to investigate task‐evoked neurotransmitter changes during sustained vigilance with differing visual input demands. Our results provide preliminary evidence that the Gabor‐based vigilance condition was associated with an increase in glutamate–glutamine (Glx) levels relative to the comparison condition, while glutathione, *N*‐acetylaspartate, and creatine levels remained stable. Importantly, traditional time‐averaged analysis did not detect significant task effects, highlighting the potential sensitivity of time‐resolved analysis. The temporal profile of Glx responses suggested gradual increases during sustained task engagement, although precise latency estimates should be interpreted cautiously given the temporal resolution of the method. Furthermore, exploratory correlation analyses indicated a positive association between Glx concentrations and behavioral performance, suggesting a potential association between visual cortical Glx dynamics and task performance during sustained vigilance. Overall, these preliminary findings suggest that dynamic Glx changes in the visual cortex may reflect combined visual processing demands and attentional‐related engagement during sustained vigilance. Importantly, these changes should not be attributed to attentional modulation alone. These findings provide preliminary support for the feasibility of time‐resolved fMRS at clinically accessible field strengths for detecting dynamic glutamatergic changes and highlight the potential utility of fMRS in elucidating task‐evoked neurotransmitter dynamics. Future studies with larger samples and more controlled experimental designs will be required to further dissociate sensory processing and attention‐related contributions.

## Graphical abstract

Using time‐resolved fMRS at 3T, we observed task‐evoked increases in Glx within the human primary visual cortex during sustained vigilance paradigm. Glx dynamics showed an exploratory association with behavioral performance. These findings suggest that time‐resolved fMRS may capture excitatory neurochemical changes related to sustained visual processing in early sensory cortex.

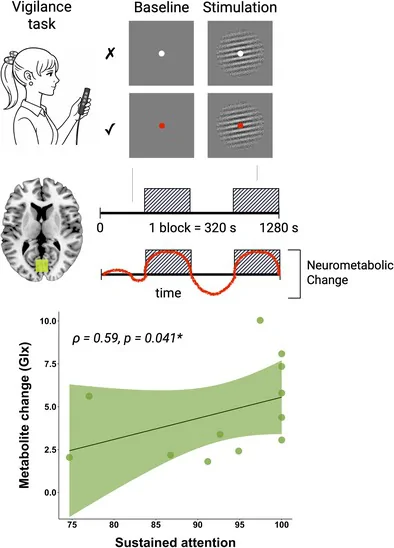

**Abbreviations**
CRLB: Cramér–Rao lower bounds
FAST (EST)MAP: fast automatic shimming technique by mapping along projections
fMRI: functional magnetic resonance imaging
fMRS: functional magnetic resonance spectroscopy
Gln: glutamine
Glu: glutamate
Glx: glutamate–glutamine
GSH: glutathione
LMM: linear mixed model
MPRAGE: magnetization prepared rapid acquisition gradient echo
NAA: *N*‐acetylaspartate
NMDA: *N*‐methyl‐D‐aspartate
PRESS: point resolved spectroscopy
SNR: signal‐to‐noise ratio
tCr: total creatine
VOI: voxel of interest

## 1 Introduction

Sustained vigilance, that is, the ability to maintain consistent attentional engagement over extended periods, is essential to effective perception and learning. Such engagement depends on coordinated activity across distributed neural systems [1, 2]. Disruptions in sustained vigilance are observed in several neurological and psychiatric disorders, including schizophrenia and attention‐deficit/hyperactivity disorder [3, 4]. These clinical manifestations underscore the importance of elucidating the specific neural and neurochemical pathways that support sustained vigilance over time. Contemporary models of attention emphasize the role of top‐down control systems, particularly within frontal and parietal cortices [5]. A growing body of magnetic resonance spectroscopy (MRS) studies has shown that glutamate–glutamine (Glx) and GABA levels in left prefrontal regions are associated with sustained attentional performance under varying task demands, highlighting the importance of excitation–inhibition balance in higher‐order control regions [6]. Together, these findings suggest that sustained vigilance is strongly associated with higher‐order executive mechanisms. However, emerging evidence from animal models suggests that sustained task engagement exerts modulatory effects even at the earliest stages of cortical processing. In macaque monkeys, attentional focus enhances the response gain of individual neurons in the primary visual cortex (V1) without altering their receptive field structure [7]. Attention also modulates contextual integration in V1 through top‐down feedback mechanisms, further supporting the potential involvement of early visual areas during sustained task engagement [8].

Multiple neurotransmitter systems contribute to sustained visual processing, including acetylcholine, which modulates vigilance and attentional focus in primate V1, dopamine, which supports executive control and reward‐based modulation, and glutamate (Glu), which mediates visual processing‐related transmission via *N*‐methyl‐D‐aspartate (NMDA) receptors, which are essential for synaptic plasticity [9, 10]. Understanding how these excitatory neurotransmitters coordinate cortical activity during sustained vigilance may help elucidate the mechanisms that support stable task engagement in dynamic visual environments. These findings suggest that early visual cortex may not function solely as a passive recipient of sensory input, but may also be influenced by sustained task demands. Theoretical accounts differ in how such modulation should be interpreted. The representation enhancement framework proposes that sustained task engagement may influence early sensory processing, potentially through changes in excitatory neural activity within the visual cortex. In contrast, information reweighting accounts propose that early sensory representations remain stable and that performance improvements arise from altered readout by higher‐order control networks [11]. Determining whether sustained task engagement is associated with neurochemical changes in the visual cortex may provide insight into these competing accounts.

Functional MRS (fMRS) detects task‐induced changes in metabolite concentrations, enabling dynamic assessment of neurometabolic processes during sensory and cognitive engagement [12, 13, 14]. Importantly, prior work has demonstrated that visual stimulation itself induces increases in glutamate concentrations in the human visual cortex, correlating with BOLD responses and the intensity and duration of visual stimuli [15, 16]. These findings highlight the role of neurometabolic plasticity in visual processing and the unique position of fMRS in investigating the biochemical mechanisms supporting early visual perception. However, whether sustained task engagement alters glutamate levels within the human visual cortex beyond such stimulus‐driven effects remains unknown. The present proof‐of‐concept study applies time‐resolved fMRS at 3T to examine whether task‐evoked neurometabolic dynamics during sustained vigilance are detectable in the visual cortex and to characterize their preliminary temporal profile. While the current design does not permit full dissociation of sensory and attention‐related contributions, the findings offer initial observations regarding the neurochemical responses of the early visual cortex under sustained vigilance. In doing so, this study highlights the potential of time‐resolved fMRS at clinically accessible field strengths as a tool for investigating task‐evoked neurometabolic dynamics during sustained vigilance and motivates future larger‐scale investigations.

## 2 Materials and Methods

### 2.1 Participants

A total of five volunteers (five females, mean age 26.5 ± 4.0 years) were recruited for the study. Visual acuity was either normal or corrected‐to‐normal based on the assessment of Tumbling E eye chart. Participants reported no history of neurological, medical, visual, or cognitive disorders, nor any contraindication to MRI. They also reported no current use of prescription medications. All participants provided written informed consent before participating in the study, which was approved by the Research Ethics Committee of National Yang Ming Chiao Tung University. All experiments were conducted following applicable guidelines and regulations.

### 2.2 fMRS Data Acquisition

**FIGURE 1:**
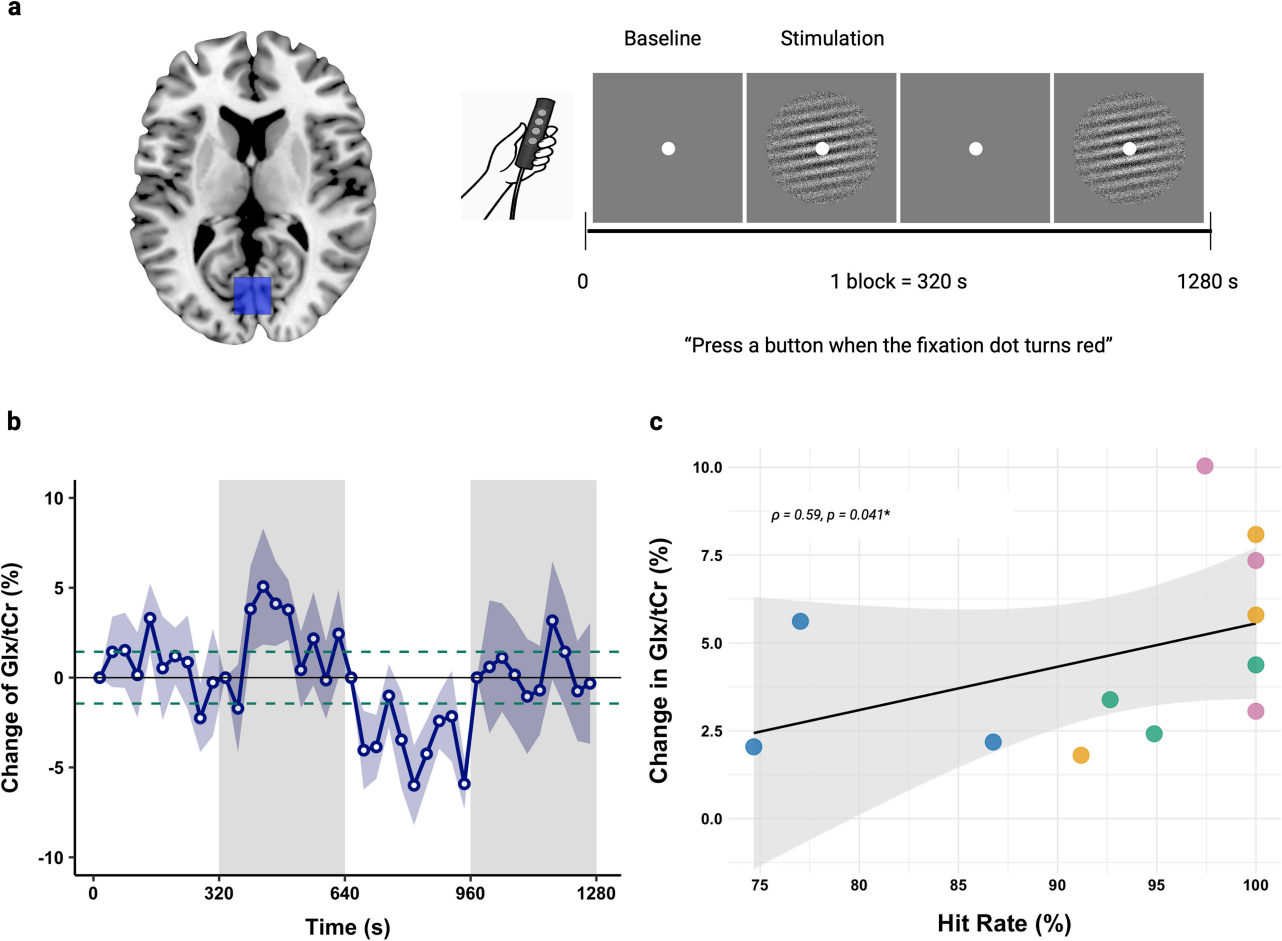
(a) fMRS voxel location and visual stimulation paradigm. The left panel displays a representative 2 × 2 × 2 cm^3^ voxel of interest (blue square) positioned over the visual cortex on a standard template. The right panel illustrates the block‐design visual stimulation paradigm, with each block lasting 320 s. Each run consisted of two baseline‐stimulation cycles, totaling 1280 s. The stimulation condition involved flashing Gabor patches, while the baseline condition featured a uniform mid‐gray screen with a central fixation dot, whose color changed to encourage vigilance and evaluate task performance. (b) Glx concentration dynamics during the task, shown in navy, are expressed as a percentage change relative to baseline levels in each block. Shaded regions around the navy line represent the standard error, and the dashed lines indicate the baseline as threshold of change for each block. Gray‐shaded blocks correspond to periods of visual stimulation. Each averaged time point represents the average of 16 TRs, aggregated across all participants. Detailed quantitative results are presented in Table 1. (c) Exploratory association between change in Glx levels and vigilance performance, measured by the vigilance task (*n* = 4; Spearman correlation coefficient *ρ* = 0.59, *p* = 0.041). Data points are color‐coded by participant. The shaded region represents the 95% confidence interval, and the solid black line indicates the trend of the dataset. * *p* < 0.05. Glx, glutamate‐glutamine; tCr, total creatine.

Each participant underwent three fMRS sessions conducted on three consecutive days at noon (15 visits). The experiments were performed using a 3.0T Siemens MAGNETOM Prisma Fit MRI scanner (Siemens Healthcare, Erlangen, Germany), equipped with a 64‐channel head matrix coil at the National Yang Ming Chiao Tung MRI Research Facility in Taiwan. Prior to the spectroscopic scans, high‐resolution T1‐weighted anatomical images were acquired using magnetization prepared rapid acquisition gradient echo (MPRAGE) sequence (192 slices, voxel size = 1 × 1 × 1 mm^3^, TR = 2500 ms, TE = 3.5 ms, flip angle = 7°, FoV = 256 mm, matrix size = 256 × 256, and bandwidth = 190 Hz/pixel) to facilitate accurate fMRS voxel positioning. Using the individual high‐resolution anatomical images, the visual voxel of interest (VOI) measuring 2 × 2 × 2 cm^3^ was manually positioned on the posterior part of the occipital lobe, covering the calcarine sulci bilaterally (Figure 1a). This careful placement of VOI was intended to ensure coverage of the central primary visual areas. Subsequent fMRS sessions replicated the VOI positioning using reference screenshots that displayed both the anatomical brain structure and the VOI position established at the baseline session. Additionally, structural images obtained in the same session as the spectroscopic data were segmented to measure the gray matter (GM), white matter (WM), and cerebrospinal fluid (CSF) content within the voxel for each participant. These tissue components were also utilized to confirm the location of the VOI across sessions. FAST (EST)MAP shimming (fast automatic shimming technique by mapping along projections) was applied to improve magnetic field homogeneity within VOI. The mean shim value, represented by the water linewidth, across participants was 13.78 ± 0.15 Hz (mean ± SEM). All fMRS scans were executed using the PRESS sequence (TR/TE = 2000/30 ms, 160 time points, and 5 min 20 s in four blocks, yielding a total acquisition time of 21.3 min). Further detailed acquisition parameters are reported in Table S2, following the minimum reporting standards for in vivo magnetic resonance spectroscopy [17].

### 2.3 Vigilance Task and Procedure

The sustained vigilance task was designed to engage a visual stimulation component to evoke activity in the visual cortex, where the fMRS VOI was positioned. The stimuli were generated utilizing MATLAB and Psychtoolbox‐3 (Brainard 1997) on a MacBook Pro workstation and were displayed on a SONY screen in the MRI room (resolution: 1920 × 1080; refresh rate: 60 Hz). Participants viewed the stimuli through a 45° angled mirror directed at a back‐projection screen (viewing distance: 282 cm).

The paradigm consisted of alternating baseline and visual stimulation blocks. During the baseline periods, a central white fixed dot was presented at the center of a gray screen within an annulus extending 0.75° eccentricity from the center. In contrast, during visual stimulation periods, oriented Gabor patches (spatial frequency of 1 cycle/degree, 100% contrast with signal‐to‐noise ratios (SNR) of 25% and 60%, Gaussian filter sigma of 2.5°, 5‐Hz flicker, 70° angle) with random spatial phases were presented within an annulus spanning 0.75°–5° from the center of a gray screen. It should be noted that the two conditions differed simultaneously in visual input characteristics, as both required sustained vigilance throughout the task. Consequently, the observed neurometabolic changes should be interpreted as reflecting task‐evoked neurochemical responses under sustained vigilance with differing visual processing demands.

Throughout the experiment, a central fixation dot remained on the screen to facilitate sustained fixation throughout the task. Participants were instructed to maintain fixation on the dot and press any button on an MRI‐safe button box when the dot randomly turned red for 250 ms approximately once every 20 s. Figure 1a shows a diagram of the visual stimuli and experimental protocol.

### 2.4 Time‐Resolved Analyses

To calculate the temporal changes in metabolite concentrations within participants, 16 consecutive time points were summed into one spectrum and then averaged across all participants to reduce noise and improve quantification accuracy. This resulted in a time course composed of 40 averaged time points (640 TRs/16 = 40) with a temporal resolution of 32 s. LCModel (Version 6.3‐1L, Copyright: Stephen W. Provencher) [18] was applied to the datasets from all participants to fit the 40 temporally averaged spectra for each participant. Metabolite concentrations were also quantified to map the time courses. Glutamate‐glutamine (Glx) levels were reported as the primary outcome, as Glx is considered a more reliable and stable metric than Glu alone in 3T proton MRS. Due to J‐coupling, the spectral peaks of Glu and glutamine (Gln) exhibit considerable overlap, resulting in mixed signals that are difficult to separate with precision [19]. Accurate quantification of Glu and Gln as distinct metabolites requires both a high SNR and a well‐defined spectral baseline, which are often not achievable in task‐based or time‐resolved acquisitions. In contrast, Glx provides greater analytical robustness and interpretive consistency, making it a more suitable indicator of task‐evoked excitatory neurotransmitter dynamics [20]. Detailed results for Glu, glutathione (GSH), *N*‐acetylaspartate (NAA) and total creatine (tCr) are also reported as secondary exploratory outcomes. The spectral range for analysis was set to 0.2–4.2 ppm. Unsuppressed water data acquired at rest prior to the task served as a reference for all quantifications.

Neurometabolic changes were expressed as percentage changes from the concentration of the first averaged time point for each participant in each block, using the equation:

$$\Delta \text{Conc}=\left(\left(C_{i}–C_{\text{1st}}\right)/C_{\text{1st}}\right)\times 100,$$

where *C*_i_ is the concentration at time point i and *C*_1st_ is the concentration at the first time point in each block for the evaluated neurotransmitter. Positive values indicate increases in neurometabolite concentration compared to baseline, while negative values indicate decreases. This produced a 5 (participants) × 40 (time points) concentration matrix per metabolite. To aid interpretation of temporal patterns at both the individual‐participant and group levels, a 3‐point moving average temporal filter was initially applied to smooth the temporal patterns prior to computing averages across participants. However, as the statistical results derived from smoothed and unsmoothed data were highly consistent, all subsequent statistical analyses and reported findings were based on the unsmoothed data for methodological consistency and transparency. The first time point of each block was used as the baseline reference to preserve temporal sensitivity in the time‐resolved analysis. While averaging multiple initial time points would provide a more stable baseline estimate, this would also reduce temporal resolution and potentially obscure early task‐evoked dynamics. This reflects an inherent trade‐off between baseline stability and temporal sensitivity in time‐resolved fMRS analyses. To assess the impact of baseline definition, an additional sensitivity analysis was performed using the average of the first two time points as the baseline reference.

### 2.5 Statistical Analysis: Metabolite Dynamics

Metabolites were quantified, and the accuracy of this quantification was assessed using the Cramér–Rao lower bounds (CRLB). Fits with CRLB > 15% were excluded from subsequent statistical analysis. The number of excluded data points per metabolite and per participant is provided in Table S3. A one‐way analysis of variance (ANOVA) was used to verify the location of GM within the VOI across sessions. Consistent with previous fMRS publications, the initial resting period was excluded from all statistical analyses due to potential familiarization effects (Ip et al. 2017).

To examine the factors associated with concentration changes while accounting for potential signal instability, a linear mixed model (LMM), defined as follows:

$$\Delta \text{Conc}∼1+\text{Task}+\mathrm{Day}+\left(1|\mathrm{Sub}\right),$$

where Δ*Conc* represents the change in metabolite concentration. *Task* and *Day* were included as categorical fixed effects. *Sub* was included as a random effect to account for intersubject variability and the nonindependence of repeated measures. Compared to a simple *t*‐test on group‐level statistics, this method offers greater statistical power, even though each participant's spectra have a lower SNR compared to the group average. Additionally, it has the potential to reveal weaker effects. Glx was specified a priori as the primary metabolite outcome based on our hypothesis regarding glutamatergic involvement in task‐evoked neurometabolic responses and was therefore not included in the multiple‐comparison correction. Glu, GSH, NAA, and tCr were treated as secondary exploratory outcomes. For each model effect, *p* values from the corresponding LMM analyses across the secondary metabolites were adjusted using the Benjamini–Hochberg false discovery rate (FDR) procedure [21]. Statistical significance was defined as a two‐sided *α* = 0.05.

To characterize the timing of threshold exceedance in the Glx time courses during sustained vigilance, we calculated the standard deviation of Δ*Conc* at the temporally averaged time points in the first block as a threshold representing the change from baseline. In the subsequent blocks, we recorded the ordinal averaged time points at which Δ*Conc* exceeded this absolute threshold for each participant. A one‐sample *t*‐test was performed on the ordinal time points at which threshold exceedance was observed across participants, with a significance level of 0.05.

### 2.6 Statistical Analysis: Correlations to Vigilance Performance

To explore the relationship between neurometabolic changes and sustained vigilance, we correlated alterations in metabolite levels with task performance. The neurometabolic changes, that is, ΔConc, were first temporally averaged in each block, and then their absolute values were calculated. Vigilance performance was assessed using hit rate, defined as the proportion of correct responses to target stimuli, that is, change in central fixation dot color, which served as our primary measure of task engagement for each participant. One participant was excluded from the correlation analysis because substantial loss of behavioral response data precluded reliable estimation of hit rate. The association between neurometabolic changes and vigilance performance was evaluated using two‐sided Spearman correlation coefficients. This analysis was considered exploratory given the small sample size, and the results should be interpreted accordingly.

## 3 Results

### 3.1 fMRS Data Quality

Individual spectra from all participants and time points were visually inspected for spectral quality, and no major artifacts were found in any of the datasets. The mean tCr linewidth was measured at 0.033 ± 0.004 ppm, with a mean SNR of 23.7 ± 2.4 per reconstructed time point. Segmentation results indicated that the average VOI comprised 69% ± 2% GM, 21% ± 4% WM, and 10% ± 3% CSF, consistent with voxel positioning across participants. Furthermore, there were no statistically significant differences in the GM fractions within the VOI over different scanning days (*F*(1, 2) = 0.288, *p* = 0.755). Spectral quality metrics per session and per participant are summarized in Table S4. Linewidth (FWHM) did not differ significantly across acquisition days (*p* = 0.288), indicating consistent spectral resolution. Although a modest effect of acquisition day was observed for SNR (*p* = 0.033), the magnitude of variation was small and remained within a comparable range across sessions.

### 3.2 Neurometabolic Functional Changes

To investigate task‐evoked neurometabolic dynamics during sustained vigilance, time‐resolved metabolite concentrations were converted into percentage changes across all participants. LMM analysis was conducted on these neurometabolic changes, with task (baseline and visual stimulus condition) and day (three consecutive midday sessions). The analysis revealed significant task effects, with a significant effect observed for Glx (*F*(1, 442) = 24.169, raw *p* < 0.001). Furthermore, a significant effect of day was observed for GSH (*F*(1, 442) = 30.423, raw *p* < 0.001), which remained significant after FDR correction across the secondary metabolites. The levels and statistical results of all metabolites are summarized in Table 1. The Glx change induced by visual stimulation at each time point was derived from averaging 16 TRs (32 s) within each participant, followed by group averaging across participants, as shown in Figure 1b. The shaded regions represent the standard error, while the dashed lines indicate the individual baseline as the threshold of change in each block.

**TABLE 1:** Relationship between change in metabolite level and the analyzed factors.

		Change of concentration				
Metabolite	CRLB (%)	Visual stimulation	Baseline state	*F* value		*p*
Glx	7.12 ± 0.95	1.18 ± 10.12	−3.31 ± 7.47	Task	24.169	0.000***
				Day	2.018	0.134
Glu	6.41 ± 0.80	1.83 ± 8.76	−1.93 ± 7.36	Task	21.072	0.000***
				Day	1.386	0.251
GSH	11.07 ± 2.94	1.16 ± 18.05	3.13 ± 17.55	Task	1.484	0.224
				Day	30.423	0.000***
NAA	2.64 ± 0.51	0.58 ± 3.29	0.18 ± 4.01	Task	1.325	0.250
				Day	0.379	0.685
Cr	10.85 ± 1.90	1.39 ± 16.34	−0.06 ± 13.62	Task	0.896	0.344
				Day	1.865	0.156

*Note:* Quantification and statistical results of each metabolite are presented, including the mean ± standard deviation of CRLB and change in metabolite concentrations. Raw *p* values are reported. Statistically significant effects are highlighted in bold. For the secondary metabolites, asterisks indicate statistical significance after FDR correction across metabolites, applied separately for each model effect (Task and Day). Glx was prespecified as the primary metabolite outcome and was not included in the FDR correction.

Abbreviations: Cr, creatine; CRLB, Cramér‐Rao lower bounds; Glu, glutamate; Glx, glutamate–glutamine; GSH, glutathione; NAA, *N*‐acetylaspartate.

***p < 0.001.

To assess the robustness of the primary finding, a sensitivity analysis was performed using the average of the first two time points as the alternative baseline reference. This approach yielded a comparable pattern of results (*F*(1, 442) = 11.85, raw *p* < 0.001), though with attenuation in effect size relative to the single time point approach, consistent with the expected trade‐off between baseline stability and temporal resolution. The corresponding statistical results after data smoothing are provided in Table S1. Furthermore, the smoothed trend line for metabolite kinetics is plotted by participant group (Figure S2) or by individual runs (Figures S4–S8).

To further evaluate the sensitivity of time‐resolved analysis for detecting task‐related changes, traditional time‐averaged spectroscopy was also analyzed, but no significant task effects were found in Glx (Figure S3). These results suggest that time‐resolved analysis may be more sensitive to transient task‐evoked metabolite dynamics than conventional time‐averaged approaches. Accordingly, the Glx dynamics observed in the present study were not detected using conventional time‐averaged analysis.

### 3.3 Correlation to Vigilance Performance

To explore the relationship between neurometabolite changes and vigilance performance during the sustained vigilance task, we analyzed the association between neurometabolite levels and behavioral performance. Participants demonstrated a mean accuracy of 90.70% ± 16.09% across stimulation conditions and days. Neurometabolite levels were expressed as the absolute changes observed within each task block, while vigilance performance was measured by task accuracy. As an exploratory analysis, Glx levels were positively correlated with vigilance performance (Spearman's *ρ* = 0.59, *p* = 0.041) (Figure 1c). A leave‐one‐subject‐out sensitivity analysis showed that the positive direction of the association remained consistent across all iterations (*ρ* = 0.492–0.627), although statistical significance was not retained after removal of any single participant. These findings should therefore be interpreted cautiously given the limited sample size.

### 3.4 Task‐Dependent Changes and Neurometabolic Response Dynamics

**FIGURE 2:**
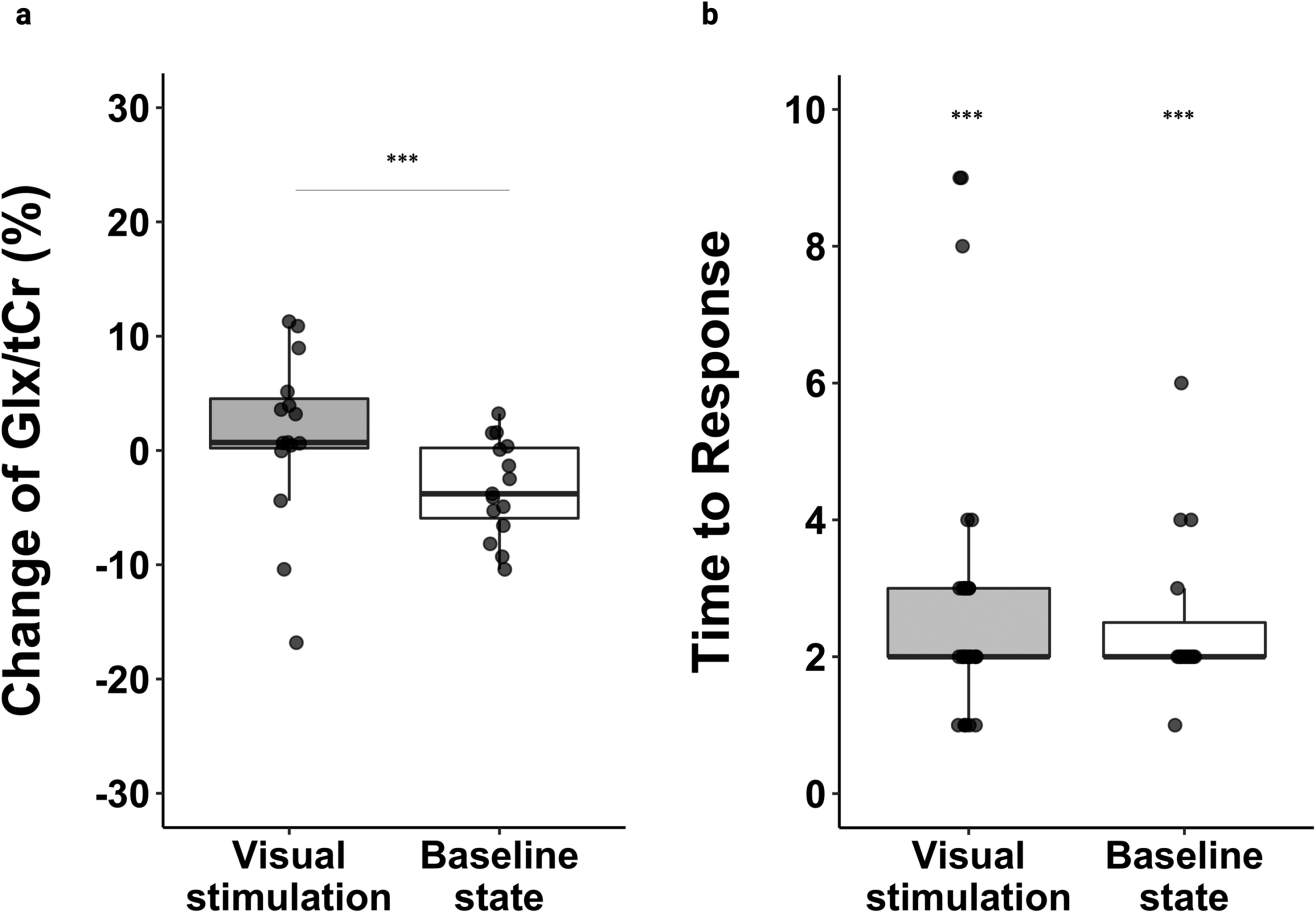
Task‐dependent changes in Glx dynamics. (a) Percentage change in Glx concentration during visual stimulation relative to the baseline state. (b) Estimated timing of threshold exceedance during visual stimulation and baseline period. ***p < 0.001. Glx, glutamate–glutamine; tCr, total creatine.

Figure 2 a presents the percentage change in Glx concentration across conditions. The LMM analysis revealed a significant main effect of task condition, confirming that Glx levels were significantly elevated during visual stimulation relative to the baseline state. On average, the task‐evoked responses exceeded the baseline threshold at approximately the second averaged time point. Within the constraints of the 32‐s temporal resolution and the threshold‐crossing approach employed, this suggests that detectable Glx changes emerged during the early phase of sustained task engagement, although the precise timing cannot be determined from the present data (estimated marginal mean visual stimulation: 92.8 s; baseline: 81.0 s). One‐sample *t*‐tests indicated that these values were statistically different from zero (visual stimulation: *t*(29) = 7.48, *p* < 0.001; baseline: *t*(14) = 7.87, *p* < 0.001), reflecting that threshold exceedance was consistently observed across participants rather than being attributable to chance (Figure 2b). The possibility of neurometabolic changes occurring prior to the detected threshold‐crossing point cannot be excluded given the temporal resolution of the acquisition. Additional results on Glu concentration changes are available in Supporting Information (Figure S1).

## 4 Discussion

This study examined whether time‐resolved fMRS at 3T could detect task‐evoked neurometabolic dynamics in the human visual cortex during sustained task engagement. Our key findings suggest that Glx levels exhibited significant increases during the Gabor‐based vigilance condition relative to the comparison condition, consistent with the involvement of excitatory neurotransmission in visual processing. Because both experimental conditions involved sustained vigilance while differing in visual input characteristics, the observed Glx changes should be interpreted as reflecting differences in visual processing demands under sustained vigilance rather than reflecting attentional modulation in isolation. Importantly, prior work by Ip et al. (2017) has demonstrated that visual stimulation itself induces glutamate increases in human visual cortex alongside BOLD responses; the present findings should therefore be considered in light of this established stimulus‐driven effect [22]. Building on this work, the present study extends this literature by examining Glx dynamics within a sustained vigilance paradigm and by exploring their association with behavioral vigilance performance. In addition, the present results highlight the potential sensitivity of time‐resolved analyses in detecting task‐evoked neurometabolic dynamics that may not be captured using conventional time‐averaged approaches. Exploratory analyses further indicated that Glx levels were positively correlated with vigilance performance. A leave‐one‐subject‐out sensitivity analysis showed that the positive association remained directionally consistent across all iterations, although statistical significance was not retained after removal of any single participant. Additionally, the temporal profile of Glx responses appeared to increase gradually over the course of sustained task engagement, though precise latency estimates should be interpreted cautiously given the 32‐s temporal resolution and the threshold‐crossing detection method employed. Metabolite changes occurring prior to the second time point cannot be excluded with the current temporal resolution.

Building on these observations, prior studies have suggested that glutamatergic neurotransmission may play a role in sustained visual processing in early sensory cortices. NMDA receptor activity, which is closely associated with Glx, has been implicated in reducing response variability and noise correlation in V1 during sustained attention tasks, supporting its relevance to excitatory signaling [9]. Other studies also indicate that attention enhances responses in V1 simple cells, increasing their sensitivity to attended stimuli and improving SNR [23]. These findings align with current models of attentional control, which propose that top‐down and bottom‐up mechanisms operate synergistically to regulate vigilance and sensory selection. Within this framework, the preliminary association between Glx levels in the visual cortex and vigilance performance observed in our study is consistent with the notion that early sensory cortices are not merely passive relays but may participate in task engagement during sustained vigilance. However, given the limited sample size and the exploratory nature of the analysis, this relationship should be interpreted with caution. The present findings do not establish a causal role of Glx in sustained visual processing, and it remains unclear whether the observed association reflects task‐specific engagement or broader task‐related neurometabolic demands. Future research integrating multimodal imaging and pharmacological manipulation could help determine whether Glx fluctuations are linked to specific attentional contributions or reflect more general neurometabolic responses.

In addition to sustained vigilance, our findings contribute to the growing body of literature linking Glx to perceptual learning and neuroplasticity. Long‐term potentiation, a fundamental mechanism underlying learning and memory, has been shown to induce synaptic plasticity in the visual cortex following visual training and high‐frequency stimulation [24, 25]. Notably, Glu levels have been associated with human visual plasticity, supporting their role in experience‐dependent cortical adaptation [26]. Pharmacological studies using NMDA antagonists such as ketamine have demonstrated impaired learning in visual discrimination tasks, underscoring the importance of glutamatergic signaling in plasticity‐related processes [27]. These earlier findings suggest that excitatory neurotransmission in the visual cortex not only contributes to visual processing but may also be involved in shaping long‐term changes in experience‐dependent plasticity. From a computational perspective on visual perception, top‐down attention may act as a gatekeeper for experience‐driven plasticity, selectively enhancing relevant inputs while suppressing irrelevant ones to prevent maladaptive learning [28]. Our preliminary data were consistent with this framework, suggesting a preliminary association between task‐evoked increases in Glx and improved behavioral performance during sustained vigilance. These observations highlight the potential of Glx as a candidate marker of task‐evoked neurocognitive processes that warrants further investigation in larger‐scale studies.

Our findings provide preliminary evidence that clinically available 3T scanners can potentially detect task‐evoked neurometabolic fluctuations, highlighting the feasibility of fMRS at 3T for investigating neurometabolic changes associated with visual processing. Previous fMRS studies at 7T have consistently reported significant increases in Glx and Glu during visual stimuli, with changes between 2% and 4% [14]. Our findings at 3T revealed comparable changes of approximately 2%, which is encouraging for translational applications, although the present sample size precludes definitive conclusions. Nevertheless, the inherent limitations of 3T, such as lower spectral resolution and reduced SNR, may result in smaller observed changes compared to 7T studies [12, 16, 29]. Additionally, variations in field strength, acquisition protocols, and baseline conditions, such as the use of fixation crosshairs on a dark screen, can influence metabolite quantification and should be carefully considered when designing future studies [30]. One of the major strengths of this study is the implementation of a dense‐sampling approach, which may enhance the precision of detecting neurotransmitter fluctuations. This method also enhanced data quality by reducing noise through repeated measures, thereby improving the stability of metabolite estimation [31, 32]. Another notable aspect of our study is the application of time‐resolved fMRS, which, unlike conventional time‐averaged analysis that integrates metabolite concentrations over extended durations, may enable the detection of transient task‐induced neurometabolic changes while providing finer temporal characterization of brain function. Previous studies at 7T have demonstrated the utility of time‐resolved fMRS in capturing neurotransmitter fluctuations during cognitive and sensory tasks [12, 15, 22]. This approach may provide greater sensitivity to subtle transient neurometabolic shifts that could be overlooked using conventional time‐averaged methods. Our study extends these methodologies to 3T and suggests that time‐resolved analysis at 3T may capture task‐evoked Glx dynamics during visual processing. The use of a block design paradigm further improved data stability and signal detection, reducing intertrial variability while maintaining experimental feasibility [33].

Additionally, our findings provide preliminary insights into the temporal dynamics of neurotransmitter responses during visual stimulation. The observed response time of Glx appeared to reach detectable levels within approximately 1–2 min, although this estimate should be interpreted cautiously given the 32‐s temporal resolution and the threshold‐crossing method employed. A previous study reported rapid Glx increases, reaching concentration peaks at 1 and 15 s after stimulus onset, implying rapid adaptation [34]. Our study, with a 32‐s time resolution, observed threshold exceedance by the second averaged time point, which may reflect methodological constraints related to acquisition and temporal averaging. Such observations may also be influenced by voxel composition, including the inclusion of both gray and WM within the VOI. These observations are consistent with earlier findings emphasizing acquisition‐related factors in the detection of glutamate fluctuations [30]. On the other hand, device specifications and targeted neurotransmitters significantly shape the observed response timing and measurement accuracy. Studies examining biexponential T2 relaxation of Glu demonstrate that field strength, time resolution, and acquisition protocols critically influence the observed dynamics [35]. For instance, in a previous fMRS study employing a temporal resolution of 2.7 min per spectrum, statistically significant glutamate changes were only detectable after approximately two acquisition points (i.e., 5.4 min), reflecting the inherent limitation imposed by longer analysis intervals [12]. Additionally, integrating fMRI‐MRS data underscores the temporal relationship between BOLD activity and Glu changes, with acquisition delays of approximately 48 s [22]. Collectively, these findings suggest that the temporal characteristics of Glx responses must be interpreted within the constraints of acquisition parameters. Within these methodological limits, our 32‐s time resolution at 3T allowed preliminary observation of task‐evoked Glx dynamics during the early phase of sustained task engagement, representing a practical balance between temporal sensitivity and spectral reliability.

In our study, NAA and tCr levels remained stable throughout the task, serving as reference metabolites relatively unaffected by the task conditions. GSH, however, fluctuated across acquisition days. While a previous study suggested that prolonged visual stimulation might elevate GSH due to increased metabolic demand, most studies have not established a link between visual stimulation and GSH levels, instead linking GSH changes more with stress responses [36]. Although some studies reported increases in lactate and decreases in aspartate levels during sensory stimulation, our data did not allow reliable quantification of these metabolites because their CRLB values exceeded our predefined threshold of 15%. While several fMRS studies have applied a 20% CRLB criterion [12, 15], we adopted a more conservative threshold to improve the reliability of neurometabolite estimation. This decision is consistent with prior reports indicating that lactate cannot be reliably quantified at 3T in healthy participants [37, 38]. Moreover, the role of aspartate in such settings is less commonly addressed in the literature, making interpretation of its potential changes uncertain.

The present study has several limitations that warrant consideration. All findings should be interpreted as preliminary observations, given the proof‐of‐concept nature of the investigation and the need for replication in larger, adequately powered samples. While the 3T scanner provided accessibility and practicality, its SNR is inherently lower than that of 7T scanners, fundamentally limiting the sensitivity to subtle and rapid neurotransmitter fluctuations. This constraint restricts the ability to achieve finer temporal resolution, potentially hindering the detection of event‐related fluctuations [39]. The current experimental design does not permit full dissociation of stimulus‐driven sensory activation from sustained visual processing, as the two conditions differed primarily in visual input characteristics while both required sustained vigilance. Future studies should incorporate visually matched control conditions to more cleanly isolate attentional contributions to Glx dynamics. Furthermore, the inclusion of only female participants was not an intentional design choice but rather a result of recruitment constraints. The visual task employed Gabor patches to engage basic visual processing in V1, and large‐scale behavioral studies have demonstrated that sex differences in low‐level visual processing, particularly contrast sensitivity and orientation discrimination, are relatively small compared with interindividual variability [40, 41]. Nevertheless, because only female participants were included, the impact of sex‐related factors on the present findings cannot be fully excluded. Therefore, the generalizability of the current findings to broader populations should be interpreted with caution. Finally, the association between Glx levels and vigilance performance was based on a limited number of observations and should be interpreted as an exploratory finding. Notably, the leave‐one‐out sensitivity analysis demonstrated that the positive association remained directionally consistent across all iterations, suggesting that the observed relationship was not driven by any single participant. While formal statistical stability cannot be established at this sample size, this pattern of results provides preliminary grounds for investigating the Glx–vigilance relationship in future, larger‐scale studies. Despite these constraints, fMRS remains one of the few noninvasive approaches capable of directly quantifying task‐evoked neurometabolic changes, offering insights into the biochemical underpinnings of neural function that are fundamentally inaccessible through hemodynamic‐based fMRI measurements. Addressing these limitations in future research could further refine the utility of dense‐sampling and time‐resolved fMRS, enhancing their applicability to both clinical and experimental neuroscience and strengthening the capacity of fMRS to answer critical questions about neurotransmitter dynamics that lie beyond the reach of conventional fMRI approaches.

## 5 Conclusion

This study provides preliminary evidence that task‐evoked Glx dynamics in the visual cortex may be associated with behavioral performance during sustained vigilance. These findings suggest that excitatory neurotransmission in early sensory areas may contribute to task‐evoked neurometabolic responses during sustained visual processing. Furthermore, our results support the feasibility of applying time‐resolved fMRS at 3T for detecting dynamic neurometabolic changes, highlighting its potential utility in cognitive and clinical neuroscience. Future studies using larger samples, higher temporal resolution, and multimodal imaging approaches will be important for further examining the relationship between neurotransmitter dynamics and cognitive processes.

## Author Contributions

I‐Ting Lee, Shang‐Hua N. Lin, Ching‐Po Lin, and Li‐Hung Chang designed the experiments. I‐Ting Lee, Rong‐Rung Kuo, and Li‐Hung Chang performed the experiments. I‐Ting Lee, Shang‐Hua N. Lin, and Li‐Hung Chang analyzed the data. I‐Ting Lee, Shang‐Hua N. Lin, and Li‐Hung Chang wrote the manuscript. All authors reviewed and approved the final version of the manuscript.

## Funding

This work was supported by the National Science and Technology Council (115‐2410‐H‐A49‐070‐MY3, 115‐2321‐B‐A49‐013, and 114‐2221‐E‐A49‐104‐MY3).

## Supporting information


**Figure S1:** (a) Glu level dynamics during the task, expressed as percentage changes relative to baseline levels in each block. A linear mixed‐model analysis was conducted on metabolic changes, with fixed effects for task (baseline vs. visual stimulation) and day (three consecutive midday sessions). This analysis revealed a significant main effect of task (*F*(1, 442) = 21.07, *p* < 0.001). Shaded region around the navy line represents the standard error, with dashed lines indicating the baseline as threshold of change for each block. Gray‐shaded blocks denote periods of visual stimulation. (b) Correlation between task‐evoked changes in Glu levels and visual attention performance in the attention task. Spearman correlation analysis indicated a nonsignificant relationship (*ρ* = 0.24, *p* = 0.45). Data points are color‐coded by participant. Shaded region around the purple line represents the 95% confidence interval, and the solid black line depicts the linear fit of the dataset. Glu, glutamate; tCr, total creatine. (c) Time of response of Glu, highlighting the timing of exceedances of the baseline threshold during the visual stimulation and baseline periods. On average, the task‐evoked responses exceeded the baseline threshold at approximately the 2nd averaged time point, indicating a reaction time of approximately one and half minute (estimated marginal mean visual stimulation: 86.4 s; baseline: 93.8 s) analyzed using one‐sample t tests (visual stimulation: *t*(29) = 7.51, *p* < 0.001; baseline: *t*(14) = 6.81, *p* < 0.001).
**Figure S2:** Smoothed trend lines of Glx and Glu level fluctuation during the task.
**Table S1:** Relationship between change in Glx and Glu concentrations after data smoothing and the analyzed factors.
**Figure S3:** Time‐averaged Glx and Glu concentrations during the attention task.
**Figure S4:** Glx response of individual participants during the attention task.
**Figure S5:** Glu responses of individual participants during the attention task.
**Figure S6:** GSH response of individual participants during the attention task.
**Figure S7:** NAA response for individual participants during the attention task.
**Figure S8:** Cr response for individual participants during the attention task.
**Table S2:** MRSinMRS checklist. Additional columns are provided for multisite or multisequence studies if necessary.
**Table S3:** Distribution of excluded data points based on CRLB threshold.
**Table S4:** Spectral quality metrics across acquisition days.

## Data Availability

The data that support the findings of this study are available on request from the corresponding author, LHC. The data are not publicly available due to restrictions imposed by the Institutional Review Board (IRB).
