# Supplementary material for: Task‐Related Neurotransmitter Dynamics in the Visual Cortex: A Proof‐of‐Concept Time‐Resolved fMRS Study Using a Sustained Vigilance Paradigm
Source: NMR Biomed. 2026 Sep 8;39(10):e70389. doi: 10.1002/nbm.70389 (PMC13553733; doi:10.1002/nbm.70389)
Supplement: Supplementary file 1 [file NBM-39-e70389-s001.docx]

**Supplementary Figure S1.** (a) Glu level dynamics during the task, expressed as percentage changes relative to baseline levels in each block. A linear mixed-model analysis was conducted on metabolic changes, with fixed effects for task (baseline vs. visual stimulation) and day (three consecutive midday sessions). This analysis revealed a significant main effect of task (*F*(1, 442) = 21.07, *p* < 0.001). Shaded region around the navy line represents the standard error, with dashed lines indicating the baseline as threshold of change for each block. Gray-shaded blocks denote periods of visual stimulation. (b) Correlation between task-evoked changes in Glu levels and visual attention performance in the attention task. Spearman correlation analysis indicated a non-significant relationship (ρ = 0.24, *p* = 0.45). Data points are color-coded by participant. Shaded region around the purple line represents the 95% confidence interval, and the solid black line depicts the linear fit of the dataset. Glu, glutamate; tCr, total creatine. (c) Time of response of Glu, highlighting the timing of exceedances of the baseline threshold during the visual stimulation and baseline periods. On average, the task-evoked responses exceeded the baseline threshold at approximately the 2nd averaged time point, indicating a reaction time of approximately one and half minute (estimated marginal mean visual stimulation: 86.4 s; baseline: 93.8 s) analyzed using one-sample t tests (visual stimulation: *t*(29) = 7.51, *p* < 0.001; baseline: *t*(14) = 6.81, *p* < 0.001).

**Supplementary Figure S2.** Smoothed trend lines of Glx and Glu level fluctuation during the task.

The lines represent percentage changes in Glx and Glu concentrations relative to the baseline level for each block. The plots were smoothed using a 3-point moving average temporal filter and subsequently averaged across all participants. Shaded region around to the navy line indicate the standard error, while gray-shaded blocks denote periods of visual stimulation. Each averaged time point reflects an average of 16 TRs, aggregated across all participants. Glx, glutamate-glutamine; Glu, glutamate; tCr, total creatine.

**Supplementary Table S1.** Relationship between change in Glx and Glu concentrations after data smoothing and the analyzed factors.

| Metabolite | Change of Concentration | |  | **F-value** | | ***P*-value** |
| --- | --- | --- | --- | --- | --- | --- |
|  | Visual Stimulation | Baseline State |  | |  | |
| Glx | 0.56±8.23 | -2.84±5.33 | Task | 21.76 | | 0.000*** |
|  |  |  | Day | 6.05 | | 0.003** |
| Glu | 1.18±7.09 | -1.86±4.64 | Task | 23.59 | | 0.000*** |
|  |  |  | Day | 7.13 | | 0.000*** |

Glx, glutamate-glutamine; Glu, glutamate.

Data were processed using a 3-point moving average temporal filter and subsequently averaged across participants. Values represent mean ± standard deviation of change in metabolite concentrations.

Statistically significant differences are highlighted in bold. **, *P* < 0.01, ***, *P* < 0.001.

 **Supplementary Figure S3.** Time-averaged Glx and Glu concentrations during the attention task.

Time-averaged concentrations of Glx and Glu are presented over the duration of the task. No significant task-related effects were observed for either Glx or Glu. Shaded region around to the navy lines indicate the standard error, while gray-shaded blocks represent periods of visual stimulation. Each averaged time point reflects the average concentration for each block, aggregated across all participants. Glx, glutamate-glutamine; Glu, glutamate.

 **Supplementary Figure S4.** Glx response of individual participants during the attention task.

The panels illustrate single-subject (S1 to S5) responses, expressed as percentage changes relative to baseline levels within each block on three consecutive days. White regions represent baseline periods, while gray area indicates periods of visual stimulation. Navy lines depict change of concentration throughout the task for each run. Glx, glutamate-glutamine.

 **Supplementary Figure S5.** Glu responses of individual participants during the attention task.

The panels illustrate single-subject (S1 to S5) responses, expressed as percentage changes relative to baseline levels within each block on three consecutive days. White regions represent baseline periods, while gray-shaded area indicates periods of visual stimulation. Navy lines depict change of concentration throughout the task for each run. Glu, glutamate.

 **Supplementary Figure S6.** GSH response of individual participants during the attention task.

The panels illustrate single-subject (S1 to S5) responses, expressed as percentage changes relative to baseline levels within each block on three consecutive days. White regions represent baseline periods, while gray-shaded areas indicate periods of visual stimulation. Navy lines depict change of concentration throughout the task for each task run. GSH, glutathione.

 **Supplementary Figure S7.** NAA response for individual participants during the attention task.

The panels illustrate single-subject (S1 to S5) responses, expressed as percentage changes relative to baseline levels within each block on three consecutive days. White regions represent baseline periods, while gray-shaded area indicates periods of visual stimulation. Navy lines depict change of concentration throughout the task for each run. NAA, N-acetylactate.

**Supplementary Figure S8.** Cr response for individual participants during the attention task.

The panels illustrate single-subject (S1 to S5) responses, expressed as percentage changes relative to baseline levels within each block on three consecutive days. White regions represent baseline periods, while gray-shaded area indicates periods of visual stimulation. Navy lines depict change of concentration throughout the task for each run. Cr, creatine.

**Supplementary Table S2.** MRSinMRS checklist. Additional columns are provided for multi-site or multi-sequence studies if necessary.

| Site (Name or Number) |  |
| --- | --- |
| 1. Hardware |  |
| a. Field strength [T] | 3 T |
| b. Manufacturer | Siemens |
| c. Model (software version if available) | Prisma (syngo MR E11) |
| d. RF coils: nuclei (transmit/ receive), number of channels, type, body part | 64-channel ^1^H head coil |
| e. Additional hardware | N/A |
| 2. Acquisition |  |
| a. Pulse sequence | PRESS |
| b. Volume of Interest (VOI) locations | visual cortex |
| c. Nominal VOI size [cm^3^, mm^3^] | 20 × 20 × 20 mm^3^ |
| d. Repetition Time (TR), Echo Time (TE) [ms, s] | TR=2000 ms, TE= 30ms |
| e. Total number of Excitations or acquisitions per spectrum  In time series for kinetic studies   i. Number of Averaged spectra (NA) per time-point ii. Averaging method (e.g. block-wise or moving average) iii. Total number of spectra (acquired / in time-series) | 160 |
| f. Additional sequence parameters  (spectral width in Hz, number of spectral points, frequency offsets)  If STEAM: Mixing Time (TM)  If MRSI: 2D or 3D, FOV in all directions, matrix size, acceleration factors, sampling method | 2000 Hz, 2048 points |
| g. Water Suppression Method | CHESS |
| h. Shimming Method, reference peak, and thresholds for “acceptance of shim” chosen | FAST(EST)MAP (Fast Automatic Shimming Technique by Mapping Along Projections) shimming was applied prior to spectroscopy acquisition |
| i. Triggering or motion correction method  (respiratory, peripheral, cardiac triggering, incl. device used and delays) | None |
| 3. Data analysis methods and outputs |  |
| a. Analysis software | LCModel 6.3 |
| b. Processing steps deviating from quoted reference or product | N/A |
| c. Output measure  (e.g. absolute concentration, institutional units, ratio)Processing steps deviating from quoted reference or product | Ratios to total creatine |
| d. Quantification references and assumptions, fitting model assumptions | Each spectrum was setting cross peak at 4.00– 0.2 ppm |
| 4. Data Quality |  |
| a. Reported variables  (SNR, Linewidth (with reference peaks)) | SNR: 24.46 ± 2.07  FWHM: 0.03 ± 0.003 ppm as reported by LCModel |
| b. Data exclusion criteria  c. Quality measures of postprocessing Model fitting (e.g. CRLB, goodness of fit, SD of residual) | SNR < 14;  FWHM > 0.05 ppm;  CRLB > 15%;  Visual inspection of fit residuals |
| d. Sample Spectrum | NA |

**Supplementary Table S3.** Distribution of excluded data points based on CRLB threshold

| Subject | Glx excluded | Glu excluded | GSH excluded | NAA excluded | Cr excluded |
| --- | --- | --- | --- | --- | --- |
| S1 | 0 | 0 | 0 | 0 | 1 |
| S2 | 0 | 0 | 21 | 0 | 3 |
| S3 | 0 | 0 | 0 | 0 | 5 |
| S4 | 0 | 0 | 0 | 0 | 0 |
| S5 | 0 | 0 | 0 | 0 | 1 |

Number of excluded data points per metabolite and per participant based on a predefined CRLB threshold of 15%. Values indicate the number of time points excluded from analysis for each metabolite.

**Supplementary Table S4.** Spectral quality metrics across acquisition days

| Day | FWHM mean ± SD | SNR mean ± SD |
| --- | --- | --- |
| 1 | 0.032 | 24.973 |
| 2 | 0.033 | 25.107 |
| 3 | 0.033 | 23.260 |

No significant differences in FWHM were observed across days, while a modest variation in SNR was detected; however, the magnitude of this variation remained within an acceptable range, indicating that overall spectral quality was not systematically affected across days.
